# Faberidilactone A, a Sesquiterpene Dimer, Inhibits Hepatocellular Carcinoma Progression Through Apoptosis, Ferroptosis, and Anti-Metastatic Mechanisms

**DOI:** 10.3390/molecules30051095

**Published:** 2025-02-27

**Authors:** Ruyu Cao, Yuhui Liu, Jiahe Bao, Mingming Rong, Jing Xu, Haibing Liao, Yuanqiang Guo

**Affiliations:** 1 State Key Laboratory of Medicinal Chemical Biology, College of Pharmacy, and Tianjin Key Laboratory of Molecular Drug Research, Nankai University, Tianjin 300350, China; 2 State Key Laboratory of Functions and Applications of Medicinal Plants, Guizhou Medical University, Guiyang 550014, China; 3 State Key Laboratory for Chemistry and Molecular Engineering of Medicinal Resources, Key Laboratory for Chemistry and Molecular Engineering of Medicinal Resources (Ministry of Education of China), Collaborative Innovation Center for Guangxi Ethnic Medicine, School of Chemistry and Pharmaceutical Sciences, Guangxi Normal University, Guilin 541004, China

**Keywords:** faberidilactone A, sesquiterpenoid dimer, STAT3, FAK, zebrafish, antitumor

## Abstract

Cancer remains a significant global public health challenge, with hepatocellular carcinoma (HCC) ranking among the top five malignancies in terms of mortality. Faberidilactone A, a sesquiterpenoid dimer isolated from *Inula japonica*, exhibits potent cytotoxicity against various human tumor cell lines and demonstrates remarkable antitumor potential. In vitro studies using HepG2 cells revealed that faberidilactone A induces apoptosis and ferroptosis, causes cell cycle arrest, enhances the production of intracellular reactive oxygen species (ROS), and disrupts mitochondrial function. Mechanistic investigations via Western blot analysis indicated that faberidilactone A impedes HepG2 cell proliferation by modulating the signal transducer and activator of the transcription 3 (STAT3) signaling pathway and inhibits metastasis by affecting the focal adhesion kinase (FAK) pathway. In vivo experiments using a zebrafish model demonstrated that faberidilactone A effectively suppresses the dissemination and metastasis of HepG2 cells and exhibits anti-angiogenic properties. When the concentration of faberidilactone A reached 10 µM, the inhibition rates of tumor proliferation, migration, and intersegmental vessels (ISVs) length were 76.9%, 72.6%, and 46.2%, respectively. These findings underscore the therapeutic potential of faberidilactone A as a promising agent for HCC treatment.

## 1. Introduction

Hepatocellular carcinoma (HCC) is a highly aggressive malignant tumor, currently ranking sixth in incidence and third in cancer-related mortality worldwide [1]. Early-stage HCC often presents with nonspecific symptoms, leading to late diagnoses when tumors are typically large and rapidly progressing, resulting in limited survival times for patients [2]. Surgical interventions remain the primary and most effective treatment modality; however, fewer than 30% of patients are eligible for surgical resection, thereby deriving direct benefit from this approach [3]. Chemoradiotherapy is another common treatment option, yet its efficacy is often compromised due to the inherent resistance of HCC cells [4]. Alternative treatments, including liver cancer ablation, hepatic artery interventions, and radiotherapy, suffer from limitations such as incomplete tumor eradication, residual cancer cells, high recurrence rates, and potential sequelae [5]. Consequently, there is an urgent need to develop effective, low-toxicity therapeutic strategies for HCC.

The exploration of bioactive natural products has emerged as a pivotal area in drug discovery, particularly for identifying monomeric compounds with significant pharmacological activities. Notable examples include artemisinin and paclitaxel, which have become cornerstone treatments in modern medicine [6,7]. The isolation, purification, and bioactivity assessment of compounds from medicinal plants represent innovative strategies for developing novel antitumor agents, thereby enhancing the utilization of natural plant resources and facilitating the discovery of biologically active lead compounds.

*Inula japonica* (*I. japonica*), belonging to the genus *Inula* L. within the Asteraceae family, is a perennial herbaceous plant predominantly found in Eastern Asia [8]. Recent studies have found that *I. japonica* contains various chemical components, including terpenoids, steroids, flavonoids, volatile oils, and polysaccharides, which have wide medicinal value [9]. Among these components, sesquiterpenoids are the most abundant characteristic constituents, such as eudane sesquiterpene, germarane sesquiterpene, guaiacane sesquiterpene, and sesquiterpene dimer, which have exhibited anti-inflammatory, antitumor, and other pharmacological activities [10,11,12,13,14]. Thus, *I. japonica* serves as a valuable source of terpenoid resources with significant research potential.

Faberidilactone A, a sesquiterpene dimer, was initially reported in 2015 following its isolation and purification from the medicinal plant *Carpesium faberi*, where it demonstrated potent inhibition of NF-κB [15]. Despite these findings, its antitumor effects and underlying mechanisms have not been thoroughly investigated. In pursuit of natural compounds with superior anticancer activity, we isolated and identified faberidilactone A from *I. japonica*, marking the first isolation of this compound within the *Inula* genus. Our preliminary assays revealed that faberidilactone A exhibits strong cytotoxicity against HepG2, MCF-7, and A549 cell lines, with efficacy comparable to etoposide. Consequently, HepG2 cells were selected for subsequent mechanistic studies. Through comprehensive in vitro and in vivo analyses focusing on apoptosis, proliferation, migration, ferroptosis, and angiogenesis, we elucidated the anti-HCC activity of faberidilactone A, thereby providing robust data to support the development of natural antitumor agents.

## 2. Results

**Figure 1:**
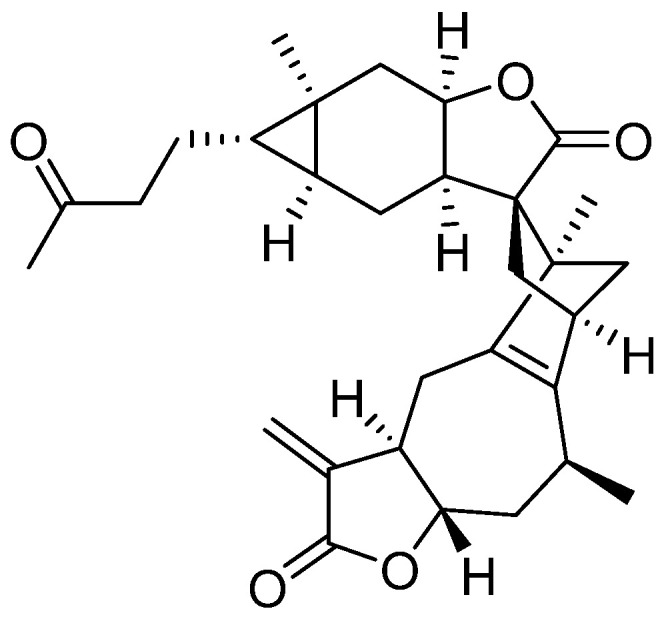
Chemical structure of faberidilactone A.

Faberidilactone A, a sesquiterpenoid dimer, was successfully isolated and purified from *I. japonica*. The compound’s ^1^H and ^13^C NMR spectra are provided in the Supplementary Material. By comparing these spectra with existing literature [15], the compound was confirmed to be faberidilactone A, as depicted in Figure 1.

### 2.1. Inhibition and Selectivity of Tumor Cell Proliferation by Faberidilactone A

The cytotoxic effects of faberidilactone A on various human tumor cell lines were evaluated using the MTT assay. The compound exhibited potent cytotoxicity against A549, HepG2, and MCF-7 cells, with half-maximal inhibitory concentration (IC_50_) values below 10 µM (Table 1). Notably, HepG2 and MCF-7 cells demonstrated higher sensitivity, with IC_50_ values of 5.4 µM for both cell lines.

Selectivity index (SI) is a powerful index to evaluate the safety and selectivity of samples [16]. Low cytotoxicity of faberidilactone A to non-malignant cell line HEK293T was detected by MTT assay, with IC_50_ value up to 61.3 ± 0.7 µM. Furthermore, the SI values of the compound against various tumor cells were calculated as follows: SI = IC_50_ (non-malignant tumor cells)/IC_50_ (tumor cells). An SI value ≥10 is considered to indicate sufficient selective inhibition of tumor cells [16]. The results demonstrated that faberidilactone A exhibited excellent safety against normal cells and high selectivity against various tumor cells, especially HepG2 and MCF-7 cells, with SI values of 11.4 (Table 2). Given that HCC is among the leading causes of cancer-related deaths globally, HepG2 cells were selected for subsequent mechanistic investigations. Based on the IC_50_ values, treatment concentrations of faberidilactone A were established for further experiments.

**Figure 2:**
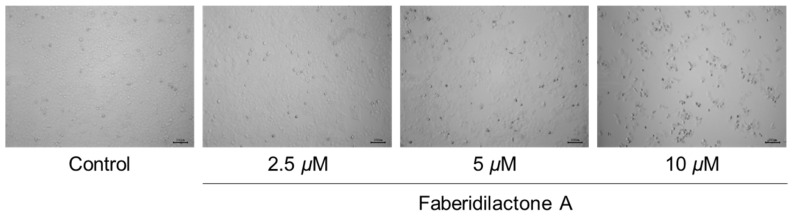
Faberidilactone A inhibits the proliferation of HepG2 cells. HepG2 cells were treated with faberidilactone A (2.5, 5, and 10 µM) for 48 h. Morphological changes in faberidilactone A-treated HepG2 cells were observed with a microscope, bar: 100 µm.

Morphological changes in HepG2 cells following faberidilactone A treatment were observed under an inverted microscope. Compared to the control group, treated cells exhibited a significant reduction in cell number after 48 h, with some cells displaying deformation, atrophy, and detachment from the culture plate. Additionally, the number of detached cells increased with higher drug concentrations (Figure 2). These observations indicate that faberidilactone A inhibits HepG2 cells proliferation in a concentration-dependent manner.

### 2.2. Induction of Apoptosis in HepG2 Cells by Faberidilactone A

**Figure 3:**
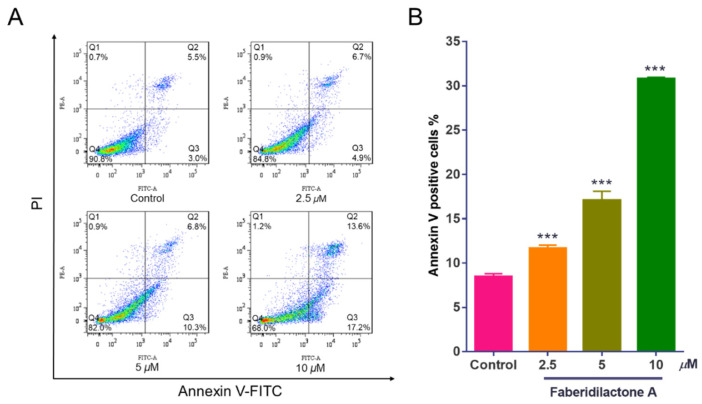
Faberidilactone A induces apoptosis in HepG2 cells. HepG2 cells were treated with different concentrations (2.5, 5, and 10 µM) of faberidilactone A for 48 h, stained with Annexin V-FITC and PI, and then the proportion of apoptotic cells was detected by flow cytometry. (**A**) Flow cytometry analysis of apoptosis in HepG2 cells. (**B**) Histogram of HepG2 apoptotic cells after 48 h of faberidilactone A treatment. All values are expressed as mean ± SD. *** *p* < 0.001 versus control group.

Apoptosis, a form of programmed cell death, is a critical mechanism for preventing cancer progression [17]. To elucidate the apoptotic effects of faberidilactone A on tumor cells, Annexin V-FITC/PI double staining was employed, followed by flow cytometry analysis. The scatter plots (Figure 3A) delineate normal cells (Annexin V^−^/PI^−^), early apoptotic cells (Annexin V^+^/PI^−^), late apoptotic cells (Annexin V^+^/PI^+^), and necrotic cells (Annexin V^−^/PI^+^). There was little change in necrotic cells represented in Q1, and the proportion of early apoptotic cells represented in Q2 increased from 3.0% (control group) to 4.9% (2.5 µM), 10.3% (5 µM), and 17.2% (10 µM). The Q3 region, which represented late apoptotic cells, showed that when the concentration of the drug reached 10 µM, the proportion of late apoptotic cells increased from 5.5% (control group) to 13.6%. The normal cells represented by Q4 decreased from 90.8% (control group) to 84.8% (2.5 µM), 82.0% (5 µM), and 68.0% (10 µM). Quantitative analysis (Figure 3B) revealed a dose-dependent increase in apoptotic cells upon faberidilactone A treatment, escalating from 8.5% in the control group to 11.7% (2.5 µM), 17.1% (5 µM), and 30.8% (10 µM). These results suggest that faberidilactone A induces programmed cell death, thereby inhibiting tumor cell proliferation.

### 2.3. Enhancement of ROS Production in HepG2 Cells by Faberidilactone A

**Figure 4:**
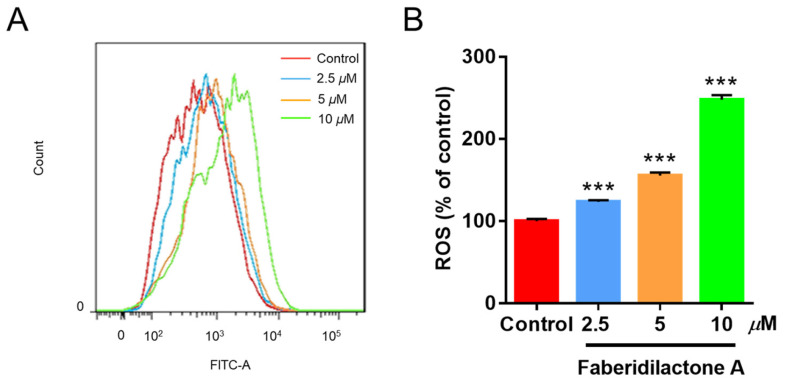
Faberidilactone A induces the accumulation of ROS in HepG2 cells. Cells were treated with different concentrations (2.5, 5, and 10 µM) of faberidilactone A for 48 h, stained with DCFH-DA, and analyzed by flow cytometry. (**A**) Flow cytometry analysis of changes in ROS content in HepG2 cells. (**B**) Histogram of relative ROS levels in HepG2 cells. All values are expressed as mean ± SD. *** *p* < 0.001 versus control group.

Reactive oxygen species (ROS) are crucial modulators of cellular growth and proliferation [18]. The intracellular ROS levels were assessed using the DCFH-DA assay, where non-fluorescent DCFH-DA is hydrolyzed to DCFH, and ROS oxidizes DCFH to fluorescent DCF, the intensity of which correlates with ROS levels. As depicted in Figure 4A, increasing concentrations of faberidilactone A resulted in a rightward shift of the fluorescence curve, indicating elevated ROS accumulation. Quantitative analysis (Figure 4B) demonstrated that ROS levels increased to 1.2-, 1.6-, and 2.5- fold in the 2.5, 5, and 10 µM treatment groups, respectively, compared to the control. This concentration-dependent rise in ROS suggests that faberidilactone A disrupts the redox balance within HepG2 cells, inducing oxidative stress and triggering apoptosis.

### 2.4. Reduction in MMP in HepG2 Cells by Faberidilactone A

**Figure 5:**
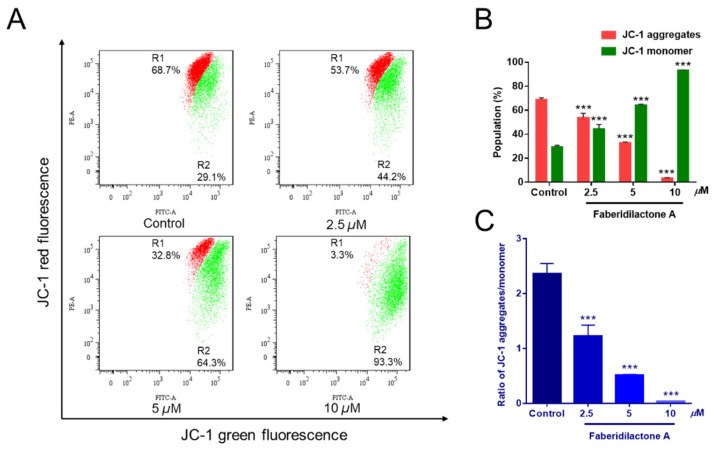
Faberidilactone A caused a decrease in MMP in HepG2 cells. Cells were treated with faberidilactone A for 48 h, stained with JC-1, and the red and green fluorescence intensity changes were detected by flow cytometry. (**A**) Flow cytometry to detect changes in MMP. (**B**) Proportion of cells containing JC-1 aggregates and JC-1 monomers. (**C**) Histogram of JC-1 aggregate/monomer proportions. All values are expressed as mean ± SD. *** *p* < 0.001 versus control group.

A decrease in mitochondrial membrane potential (MMP) is a hallmark of apoptosis [19]. To determine whether faberidilactone A-induced apoptosis is associated with MMP reduction, JC-1 staining followed by flow cytometry analysis was performed. JC-1 aggregates emit red fluorescence in healthy mitochondria, whereas JC-1 monomers emit green fluorescence in apoptotic or dysfunctional mitochondria [20]. Treatment with faberidilactone A resulted in a concentration-dependent increase in green fluorescence and a corresponding decrease in red fluorescence (Figure 5A). Quantitative analysis (Figure 5B) showed a reduction in cells with JC-1 aggregates and an increase in cells with JC-1 monomers as the drug concentration increased. Additionally, the red-to-green fluorescence intensity ratio decreased significantly with higher concentrations of faberidilactone A (Figure 5C), dropping to 51.9%, 21.6%, and 1.6% at 2.5, 5, and 10 µM, respectively, compared to the control. These findings indicate that faberidilactone A induces mitochondrial dysfunction by lowering MMP, thereby promoting apoptosis in HepG2 cells.

### 2.5. Promotion of Intracellular Lipid ROS Production in HepG2 Cells by Faberidilactone A

**Figure 6:**
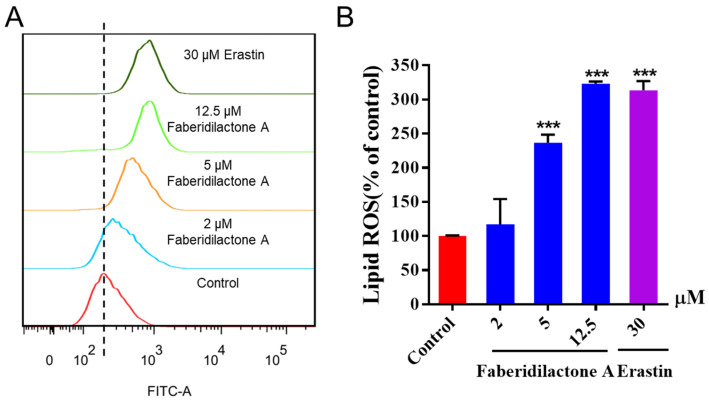
Faberidilactone A enhanced lipid ROS levels in HepG2 cells. After 48 h of treatment with different concentrations (2, 5, and 12.5 µM), cells were stained with C11 BODIPY ^581/591^ and analyzed by flow cytometry. (**A**) Flow cytometry analysis demonstrating changes in lipid ROS levels in HepG2 cells. (**B**) Histogram illustrating relative lipid ROS levels in HepG2 cells. All values are expressed as mean ± SD. *** *p* < 0.001 versus control group.

The determination of lipid reactive oxygen species (ROS) levels, a crucial marker of ferroptosis, was performed using C11 BODIPY ^581/591^ staining and flow cytometry. C11-BODIPY ^581/591^ was readily incorporated into the cell membrane and fluoresced red; however, it shifted to green upon oxidation by free radicals. As shown in Figure 6A, the horizontal axis represents the strength of the fluorescence signal, and the vertical axis represents the count of the cells. With the increase in dose concentration, the curve gradually shifted to the right, indicating that the intensity of green fluorescence increased in a concentration-dependent manner. Quantitative analysis further confirmed that the accumulation of lipid ROS increased to 1.2-, 2.4- and 3.2- fold in the 2, 5, and 12.5 µM treatment groups, respectively, compared with the control group. Notably, lipid ROS level was elevated, comparable to those of the positive control, erastin, when the concentration of faberidilactone A reached 12.5 µM (Figure 6B). During the action of faberidilactone A, apoptosis (Figure 3) was less pronounced than the cytotoxic effects shown in Table 1, suggesting that alternative modes of cell death may have occurred. The upregulation of intracellular lipid ROS levels corroborated the occurrence of ferroptosis.

### 2.6. Depletion of GSH in HepG2 Cells by Faberidilactone A

**Figure 7:**
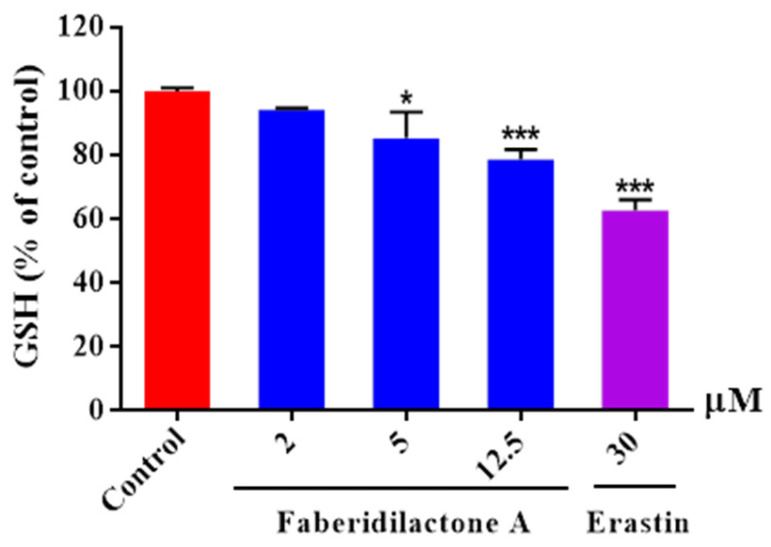
Faberidilactone A enhanced the depletion of GSH in HepG2 cells. Cells were lysed following a 24 h treatment with the drug at concentrations of 2, 5, and 12.5 µM. The absorbance of the resulting product was measured using a microplate reader, in accordance with the kit instructions. A quantitative analysis of the relative levels of GSH was performed, and all values are expressed as mean ± SD. * *p* < 0.05 and *** *p* < 0.001 versus control group.

Glutathione (GSH) is a potent endogenous antioxidant that can be enzymatically converted into its oxidized form while simultaneously reducing lipid peroxides, thereby alleviating oxidative stress damage. Compared to the control group, intracellular GSH levels in each treatment group showed a decrease. Moreover, as the concentration of the drug treatment increased, the degree of GSH depletion became more pronounced. At a concentration of 12.5 µM, the GSH content was reduced to 78.5% of its original level (Figure 7). These findings suggested that faberidilactone A exacerbated the depletion of this important antioxidant.

### 2.7. Cell Cycle Arrest Induced by Faberidilactone A in HepG2 Cells

**Figure 8:**
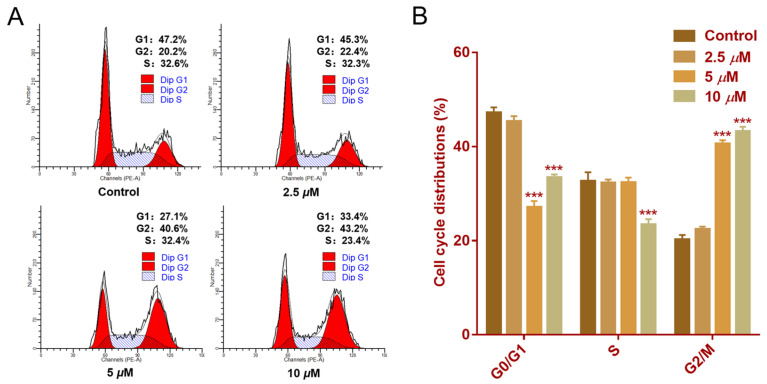
Effect of faberidilactone A on the cell cycle distribution of HepG2. HepG2 cells were treated with different concentrations (2.5, 5, and 10 µM) of faberidilactone A for 48 h. (**A**) Cell cycle distribution was analyzed by flow cytometry. (**B**) Quantitative analysis of cell cycle phases. All values are expressed as mean ± SD. *** *p* < 0.001 versus control group.

Cell cycle progression is essential for cellular proliferation [21]. Flow cytometry analysis was conducted to assess the impact of faberidilactone A on cell cycle distribution. The cell cycle profile (Figure 8A) displayed distinct peaks corresponding to the G0/G1, S, and G2/M phases. Quantitative data (Figure 8B) revealed an increase in the proportion of cells in the G2/M phase from 20.2% in the control group to 22.4% (2.5 µM), 40.6% (5 µM), and 43.2% (10 µM). Concurrently, the percentage of cells in the S phase decreased from 32.6% (control) to 23.4% (10 µM). These results suggest that faberidilactone A induces cell cycle arrest at the G2/M phase, thereby impeding HepG2 cell proliferation.

### 2.8. Modulation of Apoptosis-Related Protein Expression by Faberidilactone A

**Figure 9:**
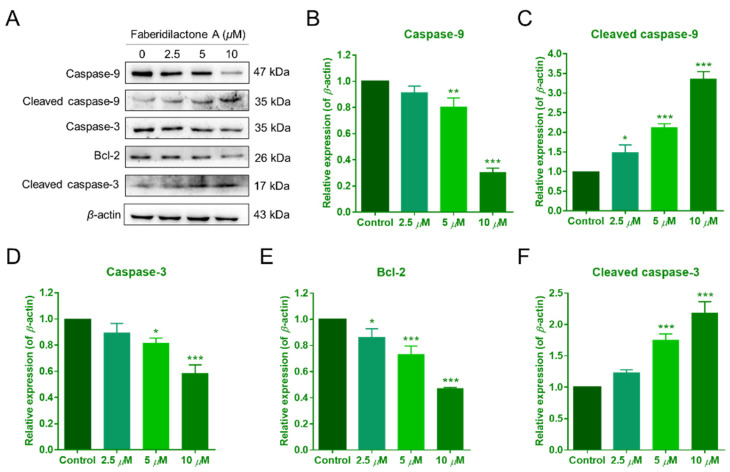
Faberidilactone A regulated the expression of apoptosis-related proteins. (**A**) Western blot analysis of Bcl-2, caspase-9, caspase-3, and their cleaved forms after treatment with faberidilactone A. (**B**–**F**) Quantitative analysis of the relative expression levels of multiple proteins. All values are expressed as mean ± SD. * *p* < 0.05, ** *p* < 0.01, and *** *p* < 0.001 versus control group.

Mitochondrial dysfunction often correlates with the activation of apoptotic signaling pathways [19]. To investigate the molecular mechanisms underlying faberidilactone A-induced apoptosis, Western blot analysis was performed to examine the expression of key proteins involved in the mitochondrial apoptotic pathway. As illustrated in Figure 9A,E, faberidilactone A treatment led to a concentration-dependent decrease in Bcl-2 expression, with a 53.0% reduction observed at 10 µM. Conversely, levels of caspase-9 and caspase-3 decreased, while their cleaved (active) forms increased in a dose-dependent manner (Figure 9B–D,F). These findings indicate that faberidilactone A downregulates anti-apoptotic proteins and activates the caspase cascade, thereby promoting mitochondria-mediated apoptosis in HepG2 cells.

### 2.9. Inhibition of HepG2 Cell Proliferation via the STAT3 Signaling Pathway by Faberidilactone A

**Figure 10:**
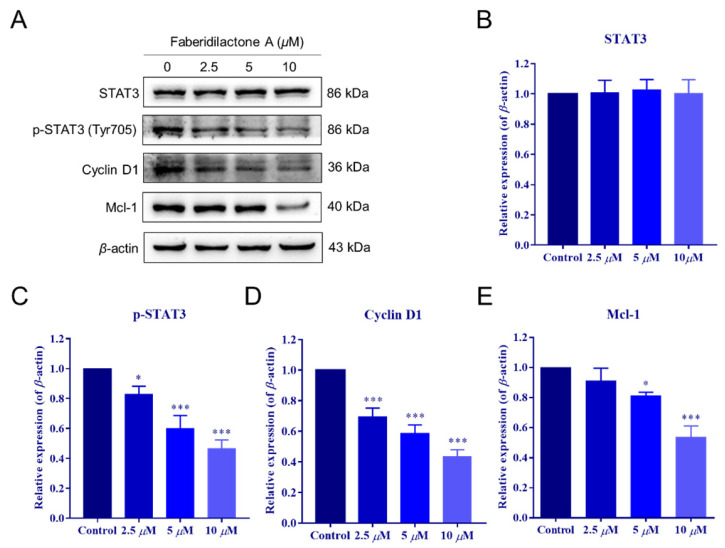
Faberidilactone A regulated the STAT3 signaling pathway. (**A**) Western blot analysis of STAT3, phosphorylated STAT3 (p-STAT3), cyclin D1, and Mcl-1 after faberidilactone A treatment. (**B**–**E**) Normalized quantification of the relative expression levels of various proteins. All values are expressed as mean ± SD. * *p* < 0.05, *** *p* < 0.001 versus control group.

Signal transducer and activator of transcription 3 (STAT3) plays a pivotal role in the proliferation and survival of various tumors [22]. To determine the involvement of the STAT3 pathway in the antitumor effects of faberidilactone A, Western blot analysis was conducted on HepG2 cells treated with 2.5, 5, and 10 µM faberidilactone A. The results demonstrated a significant, concentration-dependent reduction in STAT3 phosphorylation at Tyr705, with a 53.3% inhibition observed at 10 µM (Figure 10A,C). Notably, total STAT3 levels remained unchanged across all treatment groups (Figure 10A,B). Furthermore, downstream targets of STAT3, including cyclin D1 and Mcl-1, were markedly downregulated in response to faberidilactone A treatment (Figure 10A,D,E). These data suggest that faberidilactone A impairs HepG2 cell proliferation by inhibiting STAT3 activation and suppressing its downstream transcriptional activity.

### 2.10. Suppression of HepG2 Cell Migration and Regulation of the FAK Signaling Pathway by Faberidilactone A

**Figure 11:**
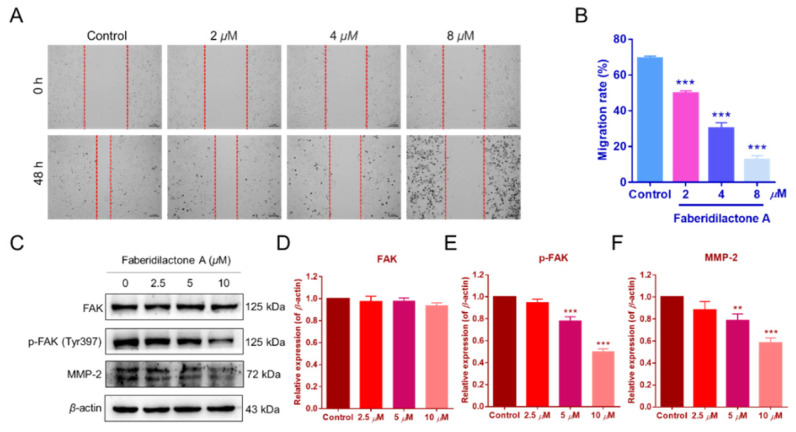
Faberidilactone A inhibited the migration of HepG2 cells and regulated the FAK signaling pathway. (**A**) Scratch assay images after faberidilactone A treatment. (**B**) Quantitative analysis of cell migration. (**C**–**F**) Western blot analysis of FAK, p-FAK, and MMP-2 expression levels. All values are expressed as mean ± SD. ** *p* < 0.01 and *** *p* < 0.001 versus control group.

Cell migration is a critical feature of tumor malignancy [23]. The impact of faberidilactone A on HepG2 cell migration was assessed using a scratch assay. Treatment with faberidilactone A resulted in a significant, dose-dependent inhibition of cell migration after 48 h (Figure 11A,B). Given the established role of the focal adhesion kinase (FAK) signaling pathway in tumor cell migration [23], Western blot analysis was performed to evaluate the expression of FAK and its phosphorylated form. While total FAK levels remained unaffected, phosphorylated FAK (p-FAK) levels decreased in a concentration-dependent manner (Figure 11C–E). Additionally, the expression of matrix metalloproteinase-2 (MMP-2), a key marker of tumor invasion and metastasis, was reduced with increasing concentrations of faberidilactone A (Figure 11C,F). These results indicate that faberidilactone A impedes HepG2 cell migration by modulating the FAK signaling pathway and downregulating MMP-2 expression.

### 2.11. Toxicity Assessment of Faberidilactone A in Zebrafish Embryos

**Figure 12:**
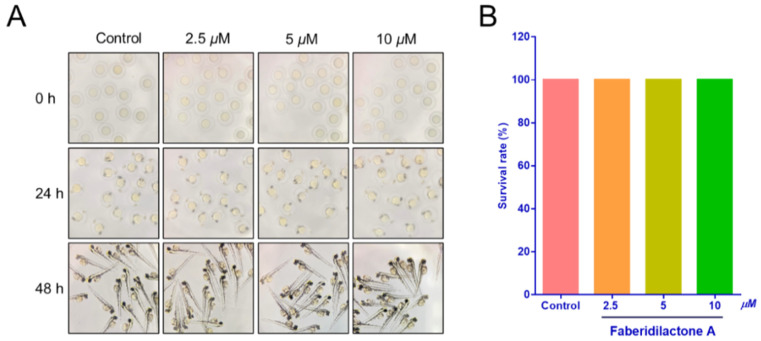
Toxicity of faberidilactone A to normal zebrafish embryos. (**A**) The growth of zebrafish embryos after 0, 24, and 48 h treatment with 2.5, 5, and 10 µM of faberidilactone A was observed via the microscope. (**B**) Statistical analysis of zebrafish survival rate after 48 h of faberidilactone A treatment.

Prior to evaluating in vivo antitumor activity, the toxicity of faberidilactone A was assessed using zebrafish embryos. Embryos at 6–9 h post-fertilization (hpf) were exposed to faberidilactone A for 48 h, and morphological changes were observed under a stereomicroscope. As shown in Figure 12A, embryos across all treatment groups displayed normal morphology, including straight spines, regular body shapes, and undisturbed yolk sacs, with no signs of deformities or tail dysplasia. Survival rates remained comparable to the control group (Figure 12B), indicating that the tested concentrations of faberidilactone A were non-toxic and suitable for subsequent in vivo experiments.

### 2.12. Inhibition of Angiogenesis in Zebrafish by Faberidilactone A

**Figure 13:**
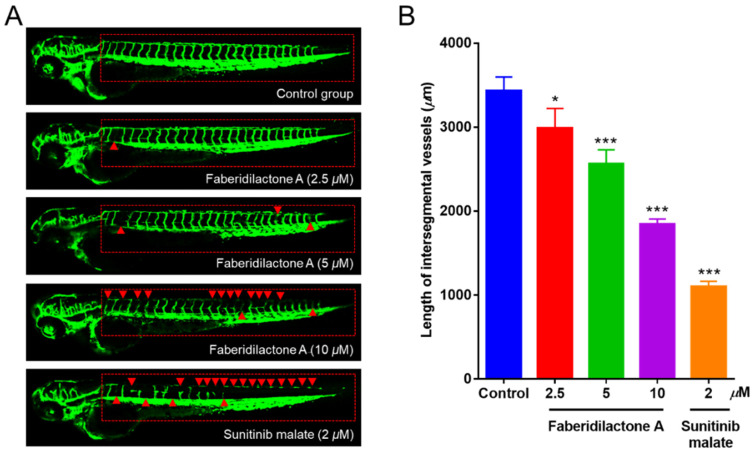
Faberidilactone A could inhibit angiogenesis in a transgenic zebrafish model. (**A**) Zebrafish embryos were treated with faberidilactone A and sunitinib for 48 h, and then the development of ISVs and DLAVs was observed under confocal microscopy. The red triangles in the figure indicated where the blood vessels were broken. (**B**) Quantitative analysis of ISVs length. All values are expressed as mean ± SD. * *p* < 0.05 and *** *p* < 0.001 versus control group.

Angiogenesis is integral to tumor growth and metastasis, providing tumors with necessary nutrients and pathways for dissemination [24]. The anti-angiogenic potential of faberidilactone A was evaluated using the transgenic zebrafish model *Tg*(*fli1:EGFP*), which was allowed for the visualization of vascular development. Treatment with faberidilactone A resulted in disrupted formation of intersegmental vessels (ISVs) and dorsal longitudinal anastomotic vessels (DLAVs), with the extent of inhibition increasing with higher concentrations (Figure 13A). Quantitative analysis using ImageJ software (version 1.51k, National Institutes of Health, Bethesda, MD, USA) confirmed a significant reduction in ISV length, with a 46.2% inhibition rate observed at 10 µM faberidilactone A (Figure 13B). These findings demonstrate that faberidilactone A effectively impairs angiogenesis in vivo, thereby potentially limiting tumor growth and metastasis.

### 2.13. Suppression of Tumor Proliferation and Metastasis in Zebrafish by Faberidilactone A

**Figure 14:**
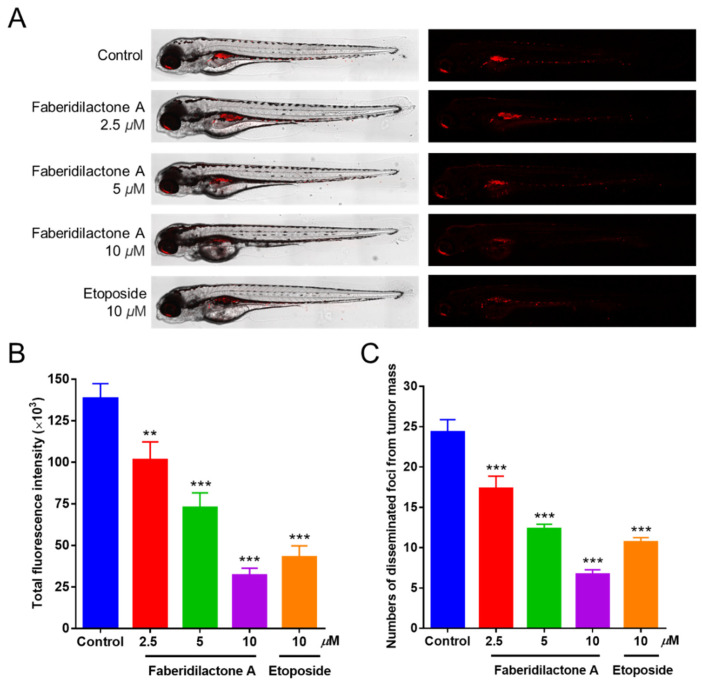
Antitumor effect of faberidilactone A in a zebrafish xenograft model. (**A**) Confocal images of HepG2 cell proliferation and metastasis after faberidilactone A treatment. (**B**) Statistical analysis of quantitative analysis of fluorescence intensity, the histogram representing the proliferative capacity of HepG2 cells. (**C**) Quantification of the number of metastasized fluorescence foci, representing the migration capacity of HepG2 cells. All values are expressed as the mean ± SD. ** *p* < 0.01 and *** *p* < 0.001 versus control group.

The in vivo antitumor activity of faberidilactone A was further evaluated using a zebrafish xenograft model. CM-DiI-labeled HepG2 cells were microinjected into the yolk sac of 48 hpf embryos, followed by treatment with non-toxic concentrations of faberidilactone A (2.5, 5, and 10 µM). Confocal microscopy imaging revealed a progressive reduction in red fluorescence intensity and the number of metastatic foci with increasing concentrations of faberidilactone A (Figure 14A). Quantitative analysis using ImageJ confirmed that faberidilactone A inhibited HepG2 cell proliferation by 26.8%, 47.5%, and 76.9% at 2.5, 5, and 10 µM, respectively (Figure 14B). Additionally, the migration inhibition rates of HepG2 cells correspondingly increased in a dose-dependent manner (Figure 14C). Notably, treatment with 10 µM faberidilactone A achieved inhibition of HepG2 cell proliferation and migration comparable to the positive control, etoposide. These results substantiate the potent antitumor efficacy of faberidilactone A in vivo.

## 3. Discussion

Hepatocellular carcinoma (HCC) is a prevalent and lethal malignant tumor characterized by rapid metastasis, aggressive progression, resistance to radiotherapy and chemotherapy, and poor prognosis [1,2]. Natural products are highly valued in anticancer therapy due to their efficacy and minimal side effects. Bioactive compounds derived from medicinal plants represent a vital source for the development of novel antitumor agents. In this study, faberidilactone A was isolated from *I. japonica* for the first time within the *Inula* genus. This sesquiterpene lactone dimer, formed through the condensation of carabane-type and guaiacyan-type sesquiterpene lactones via a Diels–Alder reaction, demonstrated significant cytotoxicity against HepG2, MCF-7, and A549 cells, warranting further investigation into its antitumor mechanisms, particularly against HCC.

Apoptosis, a programmed form of cell death, is a critical target in cancer therapy [17]. This study demonstrated that faberidilactone A induced apoptosis in HepG2 cells in a dose-dependent manner, as evidenced by morphological changes and increased Annexin V-FITC/PI staining. Apoptotic pathways can be categorized into extrinsic and intrinsic pathways, with the latter being mediated by mitochondrial dysfunction [17]. Mitochondria play a central role in cellular metabolism and apoptosis regulation [25]. Elevated ROS levels, as induced by faberidilactone A, disrupt mitochondrial homeostasis, leading to mitochondrial membrane potential (MMP) loss and subsequent apoptosis [18,26]. The JC-1 assay confirmed a significant reduction in MMP upon treatment, further supporting the involvement of the intrinsic apoptotic pathway.

The Bcl-2 family of proteins are key regulators of apoptosis, where downregulation of anti-apoptotic proteins like Bcl-2 can trigger mitochondrial outer membrane permeabilization (MOMP) and activate the caspase cascade [20,27]. The Western blot analysis revealed that faberidilactone A downregulates Bcl-2 and Mcl-1 while activating caspase-9 and caspase-3, thereby promoting apoptosis in HepG2 cells. This indicates that faberidilactone A activates the mitochondrial apoptotic pathway to exert its antitumor effects.

Ferroptosis is a promising cancer treatment strategy that primarily involves the accumulation of lipid peroxides and iron-dependent oxidative stress [19]. Lipid ROS were upregulated in a concentration-dependent manner, while GSH, a crucial antioxidant for maintaining cellular redox balance, was significantly reduced. This suggests that faberidilactone A disrupts cellular redox homeostasis by enhancing the combined effects of lipid peroxide production and the depletion of endogenous antioxidants, thereby promoting ferroptosis.

Uncontrolled cell proliferation is a hallmark of cancer, driven by dysregulated cell cycle progression [25]. Faberidilactone A was found to induce cell cycle arrest at the G2/M phase in HepG2 cells, disrupting the continuous cycle necessary for tumor cell proliferation. The STAT3 signaling pathway, crucial for tumor growth and survival, was significantly inhibited by faberidilactone A, as evidenced by reduced STAT3 phosphorylation and downregulation of downstream targets such as cyclin D1 and Mcl-1 [22,28,29]. Inhibition of STAT3 disrupts the transcription of genes involved in cell cycle progression and apoptosis, thereby inhibiting tumor cell proliferation.

Moreover, tumor metastasis involves cell migration and invasion, processes regulated by the FAK signaling pathway [23]. Faberidilactone A effectively suppressed HepG2 cell migration in vitro and downregulated phosphorylated FAK and MMP-2 expression, indicating its role in inhibiting the FAK pathway and, consequently, tumor metastasis [24,30,31,32]. Notably, reduced p-FAK levels can lead to decreased cyclin D1 stability, aligning with our observations of cyclin D1 downregulation.

In vivo studies using zebrafish models further validated the antitumor efficacy of faberidilactone A. The compound exhibited significant anti-angiogenic effects, disrupting the formation of essential blood vessels necessary for tumor growth and metastasis [24]. Additionally, in the zebrafish xenograft model, faberidilactone A inhibited both proliferation and migration of HepG2 cells, demonstrating its potential as a comprehensive antitumor agent.

## 4. Materials and Methods

### 4.1. Materials and Cell Culture

Dulbecco’s Modified Eagle’s Medium (DMEM, VivaCell) and Fetal Bovine Serum (FBS, BI, Israel) were supplied by Lab Biotech Co., Ltd. (Jinan, China). An amount of 3-(4,5-dimethylthiazol-2-y1)-3,5-di-phenyltetrazolium bromide (MTT) was purchased from BioFroxx (Guangzhou, China). Annexin V-FITC Apoptosis Assay Kit, Cell Cycle and Apoptosis Assay Kit, Reactive Oxygen Species Assay Kit, JC-1 Mitochondrial Membrane Potential Assay Kit, and BCA Protein Concentration Assay Kit were purchased from Beyotime Biotechnology Co., Ltd. (Shanghai, China). C11 BODIPY ^581/591^ was obtained from GlpBio (Shanghai, China). CheKine™ Micro Reduced Glutathione (GSH) Assay Kit was purchased from Akkbine Biotechnology Co., Ltd. (Wuhan, China). Cell tracer CM-DiI was provided by Yeasen Biotechnology Co., Ltd. (Shanghai, China). Mouse monoclonal antibodies against STAT3, caspase-9 and rabbit monoclonal antibodies against cleaved caspase-3, caspase-3, cleaved caspase-9, cyclin D1, Mcl-1, Bcl-2, phospho-STAT3 (Tyr 705), FAK, phospho-FAK(Tyr397), and β-actin monoclonal antibodies were purchased from Cell Signaling Technology (Danvers, MA, USA). Goat anti-rabbit IgG-HRP and goat anti-mouse IgG-HRP were provided by Univ Biotechnology Co., Ltd. (Shanghai, China).

HepG2, MCF-7, A549, and HEK293T cells were obtained from Shanghai Institute of Biological Sciences, Chinese Academy of Sciences (Shanghai, China). The cells were cultured in DMEM containing 10% (*v*/*v*) FBS and 100 U/mL penicillin/streptomycin in a water-saturated atmosphere with 95% air and 5% CO_2_. Adult AB strains of wild-type and genetically modified zebrafish were purchased from Feixi Biotechnology Co., Ltd. (Shanghai, China).

### 4.2. Extraction and Purification of Faberidilactone A

The protocols for extracting, isolating, and purifying faberidilactone A from *I. japonica*, as well as the NMR results, are presented in the Supplementary Materials.

Faberidiactone A: ^1^H NMR (400 MHz, CD_3_OD): *δ*_H_ 0.45 (1H, m, H-1), 1.53 (2H, m, H-2), 2.57 (2H, m, H-3), 0.30 (1H, m, H-5), 1.73 (1H, m, H-6a), 0.67 (1H, m, H-6b), 2.24 (1H, m, overlapped, H-7), 4.89 (1H, m, H-8), 2.30 (1H, m, overlapped, H-9a), 0.88 (1H, m, H-9b), 2.08 (1H, m, overlapped, H-13a), 1.68 (1H, dd, *J* = 11.8, 3.0 Hz, H-13b), 1.09 (3H, s, H-14), 2.15 (3H, s, H-15), 2.62 (1H, m, overlapped, H-2′), 2.12 (1H, m, H-3′a), 1.22 (1H, m, overlapped, H-3′b), 2.99 (1H, m, overlapped, H-6′a), 2.01 (1H, m, overlapped, H-6′b), 2.59 (1H, m, overlapped, H-7′), 4.34 (1H, m, H-8′), 2.21 (1H, m, overlapped, H-9′a), 1.98 (1H, m, H-9′b), 2.96 (1H, m, overlapped, H-10′), 6.15 (1H, d, *J* = 2.2 Hz, H-13′a), 5.71 (1H, d, *J* = 2.2 Hz, H-13′b), 1.22 (1H, d, *J* = 7.0 Hz, H-14′), 1.33 (3H, s, H-15′); ^13^C NMR (100 MHz, CD_3_OD): *δ*_C_ 34.7 (C-1), 24.5 (C-2), 44.3 (C-3), 211.9 (C-4), 24.4 (C-5), 29.1 (C-6), 41.6 (C-7), 77.6 (C-8), 38.7 (C-9), 17.1 (C-10), 65.3 (C-11), 184.4 (C-12), 39.0 (C-13), 18.5 (C-14), 30.0 (C-15), 153.5 (C-1′), 49.7 (C-2′), 53.2 (C-3′), 57.3 (C-4′), 139.8 (C-5′), 28.3 (C-6′), 49.2 (C-7′), 83.2 (C-8′), 40.2 (C-9′), 33.8 (C-10′), 141.7 (C-11′), 172.1 (C-12′), 119.8 (C-13′), 20.6 (C-14′), 15.2 (C-15′).

### 4.3. Cell Morphology Observation and Toxicity Detection

MTT method was used to detect the effect of faberidilactone A on the viability of various tumor cells [33,34,35]. The specific experimental process is described in the Supplementary Materials.

### 4.4. Apoptosis Assays

The apoptosis of HepG2 cells treated with different concentrations of faberidilactone A was analyzed by Annexin V-FITC/PI double staining [33,34,35]. The specific details of the experimental method are described in the Supplementary Materials.

### 4.5. Determination of Intracellular ROS

To investigate the effect of faberidilactone A on the intracellular ROS content, cells were labeled with DCFH-DA probe, and the changes in fluorescence intensity were detected via flow cytometry [33,34,35]. Experimental method details are presented in the Supplementary Materials.

### 4.6. Determination of MMP

Treated with different concentrations of faberidilactone A, HepG2 cells were labeled with JC-1 probe, and the changes in MMP were analyzed by flow cytometry [33,34,35]. Details of the specific experiments are shown in the Supplementary Materials.

### 4.7. Lipid ROS Detection

The C11 BODIPY ^581/591^ probe was used to monitor lipid ROS accumulation within cells [36,37]. The details of the experiments are shown in the Supplementary Materials.

### 4.8. GSH Assay

Cells were processed according to the instructions to prepare samples, and then GSH levels were measured with the kit. The specific methods are demonstrated in the Supplementary Materials.

### 4.9. Cell Cycle Arrest Assay

The effect of faberidilactone A on the cell cycle distribution of HepG2 cells was examined using the Cell Cycle and Apoptosis Assay Kit [33,34,35]. Experimental methods are provided in the Supplementary Materials.

### 4.10. Wound Healing Assay

The effect of faberidilactone A on the migration ability of HepG2 cells was detected by wound scratch assay [33,34,35], and the specific method is presented in the Supplementary Materials.

### 4.11. Western Blotting Analysis

Western blotting assay was used to analyze the effect of faberidilactone A on the expression of related key proteins [33,34,35]. Experimental methods are presented in the Supplementary Materials.

### 4.12. In Vivo Viability Assays

All procedures involving animals were approved by the Institutional Animal Protection Committee of Nankai University (No.2021-SYDWLL-000225). According to the supplier’s guidance, the cultivation of zebrafish and the acquisition of embryos for experiments were carried out in accordance with scientific methods [33,34,35], which are shown in the Supplementary Materials.

#### 4.12.1. Toxicity Testing of Zebrafish Embryos

The non-toxic concentration of faberidilactone A was determined based on the survival of zebrafish at different concentrations for subsequent experiments. The specific steps are provided in the Supplementary Materials.

#### 4.12.2. In Vivo Antitumor Experiments in Zebrafish Xenograft Models

In order to analyze the effect of faberidilactone A on fluorescence intensity and metastasis in zebrafish xenograft models, antitumor experiment was carried out in zebrafish [33,34,35]. Experimental methods are provided in the Supplementary Materials.

#### 4.12.3. Anti-Angiogenic Experiments in Zebrafish In Vivo

The inhibitory effect of faberidilactone A on angiogenesis in zebrafish was evaluated by transgenic zebrafish *Tg*(*fli1:EGFP*) model [33,34,35]. Details of the method are described in the Supplementary Materials.

### 4.13. Statistical Analysis

GraphPad Prism 7.0 software was used to analyze the experimental data. All data were presented as the mean ± SD. One-way ANOVA was used to compare significant differences between groups. Probabilities (P) of less than 0.05 indicated a significant difference.

## 5. Conclusions

Natural products continue to be a rich source of therapeutic agents for cancer prevention and treatment. In this study, faberidilactone A was isolated from *I. japonica* and evaluated for its antitumor properties. The compound effectively induced apoptosis through ROS-mediated mitochondrial dysfunction, drove ferroptosis by aggravating GSH depletion and lipid peroxide accumulation, inhibited cell proliferation through causing G2/M cell cycle arrest and suppressing the STAT3 signaling pathway, and prevented cell migration by modulating the FAK pathway. In vivo studies using zebrafish models confirmed its anti-angiogenic and antitumor activities, demonstrating its ability to inhibit tumor proliferation and metastasis. These findings highlight faberidilactone A as a promising candidate for the development of novel therapeutic agents against HCC, offering new avenues for effective and targeted cancer treatment.

## Figures and Tables

**Table 1:** Toxic activity of faberidilactone A against three human tumor cell lines.

Compound	IC_50_ (µM)		
	A549	HepG2	MCF-7
**Faberidilactone A**	8.4 ± 0.3	5.4 ± 0.5	5.4 ± 0.4
**Etoposide ^a^**	15.3 ± 1.9	3.6 ± 0.1	17.1 ± 1.1

^a^ Positive control. All results are presented as mean ± SD.

**Table 2:** Selectivity of faberidilactone A against three human tumor cell lines.

Compound	SI ^a^		
	A549	HepG2	MCF-7
**Faberidilactone A**	7.3	11.4	11.4

^a^ Selectivity index. SI = IC_50_ (non-malignant tumor cells)/IC_50_ (tumor cells).

## Data Availability

Data will be made available on request.

## Supplementary Materials

The following supporting information can be downloaded at: www.mdpi.com/article/10.3390/molecules30051095/s1.
