# Supplementary material for: Faberidilactone A, a Sesquiterpene Dimer, Inhibits Hepatocellular Carcinoma Progression Through Apoptosis, Ferroptosis, and Anti-Metastatic Mechanisms
Source: Molecules. 2025 Feb 27;30(5):1095. doi: 10.3390/molecules30051095 (PMC11901444; doi:10.3390/molecules30051095)

Supplementary material for

Faberidilactone A, a Sesquiterpene Dimer, Inhibits Hepatocellular Carcinoma Progression through Apoptosis, Ferroptosis, and Anti-Metastatic Mechanisms

Contents

1. Extraction, isolation, and purification of faberidilactone A

2. MTT assay

3. Apoptosis assay via flow cytometry

4. ROS generation assay

5. MMP assay

6. Lipid ROS assay

7. GSH assay

8. Cell cycle assay

9. Wound-scratch assay

10. Western blotting analysis

11. Rearing and embryo acquisition of zebrafish

12. Evaluation of toxicity in zebrafish embryos

13. *In vivo* anti-tumor experiments in zebrafish xenograft models

14. Anti-angiogenetic experiments in zebrafish *in vivo*

15. NMR spectra of faberidilactone A

1. Extraction, isolation, and purification of faberidilactone A

The plant *Inula japonica* (*I. japonica*) was purchased in Henan Province in October 2020 and stored after successful identification. The dried flowers of this plant (10.0 kg) were heated and refluxed three times with 95% methanol to obtain the residue (1.8 kg), which was then refluxed under reduced pressure to obtain the crude extract. Then, the extract was suspended in H_2O and partitioned with petroleum ether (PE) and ethyl acetate (EtOAc). After standing for stratification, the upper solution was concentrated to obtain the PE-soluble portion (193.0 g). The extract was placed in a silica gel column (200–300 mesh), eluted by a gradient of PE-acetone (v/v) in different ratios (100:0 to 100:35), and used thin layer chromatography (TLC) for monitoring, resulting in 9 components (Fr.1–Fr.9). Furthermore, Fr.5 (5.6 g) was separated by an eluate containing 83% MeOH of H_2O using medium pressure liquid chromatography (MPLC) to obtain two components ($F_{5\text{-}1}$ and $F_{5\text{-}2}$). Finally, $F_{5\text{-}2}$ was purified via preparative high performance liquid chromatography (prep-HPLC, YMC-pack ODS-AM column, 20 mm × 250 mm) to obtain colorless oil (28.0 mg) with an eluent of 81% MeOH in H_2O, which is faberidilactone A. The extraction yield was calculated to be 0.00028%.

2. MTT assay

HepG2 cells were seeded in a 96-well plate at a cell density of 5000 cells/well and then cultured for 24 h. Different concentrations of DMSO-dissolved faberidilactone A were added to the medium and cultured for 48 h. Morphological changes of the cells were observed and recorded under a microscope. 20 μL of MTT (5 mg/mL) was added to each well and incubated at 37°C for 4 h before the liquid in the wells was discarded. Then 150 μL of DMSO was added, and OD values were measured at 492 nm using a microplate reader (Thermo Fisher Technologies, USA). The cell inhibition rate (%) of the compound was determined according to the following formula, and the half maximal inhibitory concentration (IC_{50}) value of the compound was calculated.

$$\text{Inhibition rate}(\%) = \frac{\text{OD}_{\text{control}} - \text{OD}_{\text{treatment}}}{\text{OD}_{\text{control}}} \times 100$$

3. Apoptosis assay via flow cytometry

During the exponential growth phase of the cells, a 12-well plate (1×10^5 cells/well) was laid. After 24 h of incubation in the incubator, different concentrations of samples were treated for 48 h. Cells were collected in centrifuge tubes, washed with pre-chilled PBS, resuspended with fresh $1\times$binder, and then passed through a cell sieve of 200 mesh and aliquoted into flow cytometry tubes. Each tube of cells was then stained with 5 μL of Annexin V-fluorescein isothiocyanate (FITC), shaken well and continued to add 10 μL of propidium iodide (PI), then incubated at 25 °C in the dark for 15 min, and detected with BD LSRFortessa Flow Cytometer (BD Biosciences). The data were processed using FlowJo flow cytometry analysis software (FLOWJO LLC, Ashland, OR, USA).

4. ROS generation assay

A 12-well plate (1×10^5 cells/mL) was seeded with a cell suspension prepared from HepG2 cells and cultured for 24 h. After 48 h of treatment with 2.5, 5, and 10 μM of faberidilactone A, the cells were collected and resuspended with 10 μM DCFH-DA probe working solution. After incubating at 37 °C for 20 min in the dark for successful staining, the cells were washed with DMEM and immediately detected with the BD LSRFortessa Flow Cytometer. FITC channels were selected to detect the stained cells, and the data were processed via FlowJo software.

5. MMP assay

HepG2 cells were laid out in a 12-well plate at a density of 1×10^5 cells/well. After 24 h of incubation in a CO_2 incubator, the cells were treated with 2.5, 5, and 10 μM of faberidilactone A for 48 h, respectively. Next, the cells were collected into centrifuge tubes, and the excess culture was discarded by centrifugation. After washing with pre-chilled PBS, the cells were suspended in 500 μL of JC-1 staining solution. Cells samples were incubated at 37 °C for 20 min, washed with PBS,

resuspended, and aliquoted into flow cytometry tubes wrapped in tin foil, and analyzed on machine. Finally, the percentage of cells containing JC-1 monomers and aggregates was analyzed with FlowJo flow analysis software.

6. Lipid ROS assay

A 12-well plate (1×10^5 cells/well) of HepG2 cells was plated and incubated for 24 h, followed by 2, 5, and 12.5 μM of faberidilactone A, respectively. Next, the cells were incubated in the incubator for 48 h and collected, then resuspended and incubated with 800 μL of 10 μM probe working solution at 37 °C for 15 min. Pre-chilled PBS-washed cells were immediately detected on a flow cytometer, and the data were quantified with FlowJo flow analysis software.

7. GSH assay

HepG2 cells in the exponential growth phase were incubated in a 6-well plate (2×10^5 cells/well) for 24 h in the incubator, and then stimulated with 2, 5, and 12.5 μM of drugs for 24 h. According to the instructions, the collected groups of cells were completely lysed with Extraction Buffer at low temperature. After centrifugation, part of the supernatant was measured by BCA Protein Assay Kit (Beyotime, P0012S) to obtain the protein concentration. Meanwhile, 20 μL was piped from the other part of the supernatant, as 140 μL of Assay Buffer and 40 μL of Chromogen were added, and then the absorbance was measured at 412 nm via a microplate reader after incubation at 25 °C in the dark for 2 min. The absorbance data is substituted into the standard curve to obtain the glutathione (GSH) content, which is then corrected with protein concentration to obtain the relative levels.

8. Cell cycle assay

HepG2 cells were planted in a 12-well plate (1×10^5 cells/mL), and subsequently incubated at 37°C for 24 h. Faberidilactone A was prepared at concentrations of 2.5, 5, and 10 μM, respectively,

and then added to the corresponding wells for 48 h. Cells were resuspended with 70% ethanol and fixed overnight in a 4 °C freezer. The next day, the ethanol in the cells was discarded and washed by centrifugation. Each group of cells was resuspended with 25 μL of 20× staining solution, 500 μL of buffer, and 10 μL of 50× RNase A (Beyotime, C1052), and then incubated at 37°C in the dark for 30 min. Finally, the samples were transferred to flow cytometry tubes and analyzed by flow cytometry. The obtained data were analyzed using ModFitLT software.

9. Wound-scratch assay

HepG2 cells were seeded in a 6-well plate with a density of 5×10^5/mL and incubated in a CO_2 incubator for 24 h until the cells were almost fully covered. Next, the cells in the plate were scraped with the tip of a sterile pipette to form cell scratches of the same width, and then the shed cells are washed off. Samples of different concentrations (2, 4, and 8 μM) were added, and scratch pictures were observed under the microscope at 0 h and 48 h, respectively. The scratch areas were quantified using ImageJ software, and the mobility of the cells was calculated.

10. Western blotting analysis

A 6-well plate was plated with well-growing HepG2 cells and cultured at 37°C for 24 h, followed by the addition of samples at concentrations of 2.5, 5, and 10 μM. After incubation for 48 h, the cells were washed with pre-chilled PBS, lysed with RIPA lysis buffer. The supernatant obtained after vortexing and centrifugation was the total protein.

The protein concentration was determined by the BCA method, from which the loading volume was calculated. SDS-PAGE gel with appropriate concentration according to protein molecular weight was prepared and the sample was added after its coagulation. Electrophoresis was stopped when the bromophenol blue indicator almost reached the bottom of the gel, and then the target protein on the gel plate was transferred to an activated PVDF membrane. After transference, the PVDF membrane

was blocked with skimmed milk powder, incubated overnight with the corresponding primary antibody in a 4°C freezer, and then incubated with a secondary antibody. After several washes with TBST, the membrane was finally immersed in ECL luminescence solution, exposed, and developed in a Tanon 5200 Multi chemigraphic luminometer. The development was recorded and processed with ImageJ software.

11. Rearing and embryo acquisition of zebrafish

During the culture process, the temperature of the zebrafish recirculating aquaculture system was controlled at about 28 °C. The light cycle was 14 h of light/10 h of darkness and sterilized using 21 W UV light. When embryos were needed, 1 adult female and 2 adult males were placed in a tank and separated with a partition. Next, the tanks were incubated overnight in a 28.5 °C thermostatic biochemical incubator (Tianjin, China), sheltered from light. The next morning, the clapboard was removed, waited about 1 h, and then embryos were collected after 30–60 min of light. Dead or unfertilized embryos were then picked out in time, and healthy embryos were placed in sterilized Holt buffer and cultured in biochemical incubators.

12. Evaluation of toxicity in zebrafish embryos

Approximately, faberidilactone A was diluted to multiple concentrations with Holt buffer. Healthy embryos at 6–9 h post-fertilization (hpf) were randomly grouped and transferred to a 12-well plate of 20–30 embryos. The zebrafish were treated with 2.5, 5, and 10 μM faberidilactone A for 48 h, during which the development of zebrafish was observed every 24 h, and the pictures of zebrafish were taken at 0, 24, and 48 h after dosing treatments. By recording the mortality of the zebrafish, the concentration of subsequent *in vivo* experiments could be determined.

13. *In vivo* anti-tumor experiments in zebrafish xenograft models

First, the embryos were obtained by mating adult wild-type zebrafish of the AB strain, and artificial

demembranation was carried out the next night. When the embryos had grown to 48 hpf, they were anesthetized. HepG2 cells at the exponential growth stage were collected and resuspended with 950 μL DMEM, added with 2 μL CM-DiI, incubated at 37 °C for 5 min, and then incubated at 4 °C for 15 min. After washing and counting, the cells were resuspended with a suitable volume of DMEM to adjust the cell density to 1×10^7 cells/mL, and 5 nL CM-DiI fluorescently labeled tumor cells were injected into the yolk sacs of the embryos. The juveniles were treated with different concentrations of drugs at 24 h after injection, and the tumor development was observed and recorded by laser confocal microscopy (Leica, Germany) after 48 h of culture. The fluorescence intensity and metastasis in zebrafish *in vivo* were quantified by ImageJ software.

14. Anti-angiogenetic experiments in zebrafish *in vivo*

Transgenic zebrafish *Tg*(*fli1: EGFP*) was employed to obtain vascular transgenic zebrafish embryos. When the embryos grew to 6 hpf, they were randomly transferred to a 12-well plate, treated with different concentrations (2.5, 5, and 10 μM) of compounds, and cultured in a zebrafish biochemical incubator for 48 h, during which time the embryos were observed and unhealthy embryos were singled out. After 48 h, the embryos were anesthetized and fixed onto agar glue, and then the breakage of intersegmental vessels (ISVs) and dorsal longitudinal anastomotic vessels (DLAVs) was observed by SP8 confocal microscopy, and the lengths of ISVs and DLAVs were counted using ImageJ software to evaluate the effect of faberidilactone A on angiogenesis.

15. NMR spectra of faberidilactone A

Figure S1. ^{1}H NMR spectrum of faberidilactone A

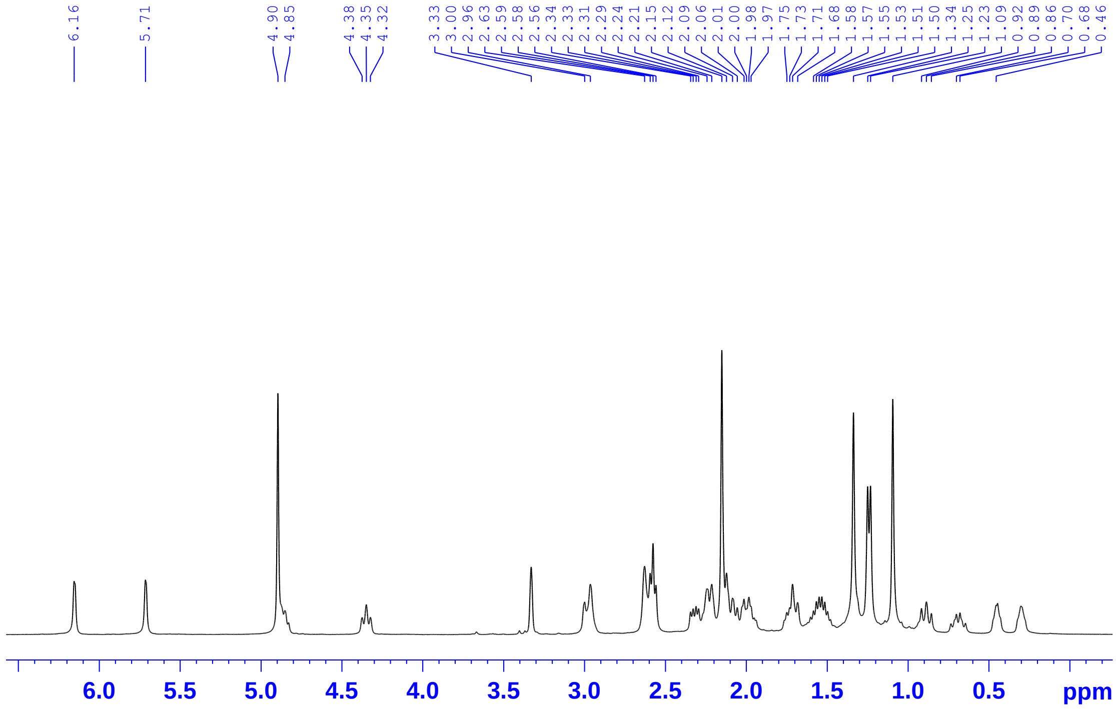

Figure S2. ^{13}C NMR spectrum of faberidilactone A

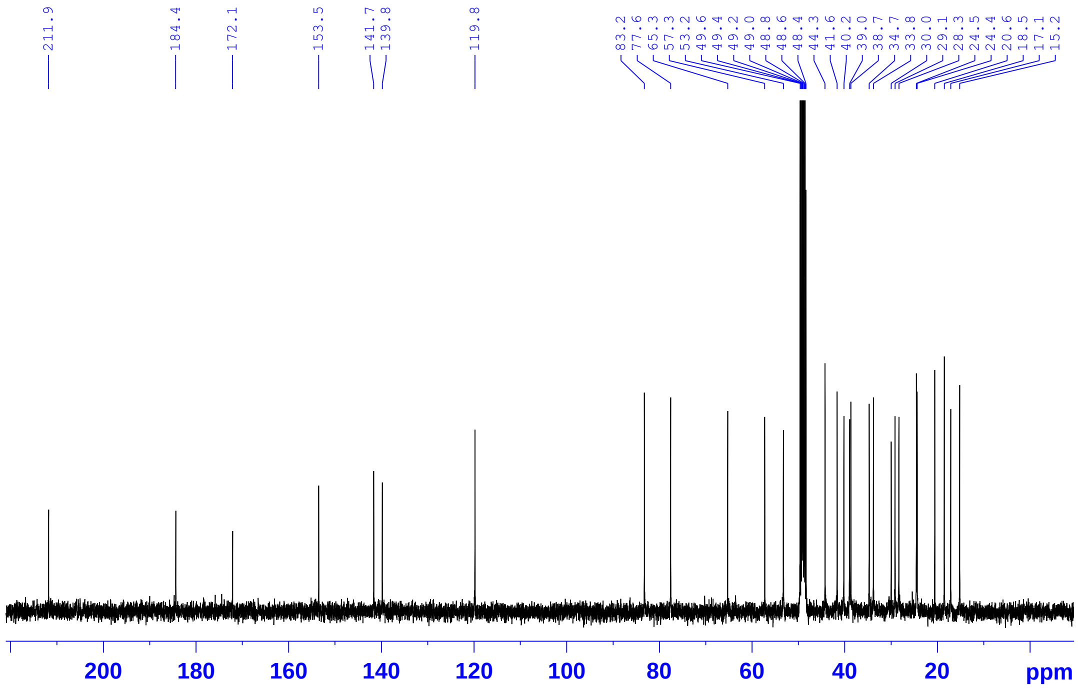

Supplement: Supplementary file 1 [file molecules-30-01095-s001.zip › molecules-3420268-supplementary.pdf]
